# MHD Stagnation Point on Nanofluid Flow and Heat Transfer of Carbon Nanotube over a Shrinking Surface with Heat Sink Effect

**DOI:** 10.3390/molecules26247441

**Published:** 2021-12-08

**Authors:** Mohamad Nizam Othman, Alias Jedi, Nor Ashikin Abu Bakar

**Affiliations:** 1 Department of Mechanical and Manufacturing Engineering, Faculty of Engineering and Built Environment, Universiti Kebangsaan Malaysia, Bangi 43600, Malaysia; P102090@siswa.ukm.edu.my; 2 Centre for Automotive Research (CAR), Faculty of Engineering and Built Environment, Universiti Kebangsaan Malaysia, Bangi 43600, Malaysia; 3 Institute of Engineering Mathematics, Faculty of Applied and Human Sciences, Universiti Malaysia Perlis, Arau 02600, Malaysia; ashikinbakar@unimap.edu.my

**Keywords:** MHD stagnation flow, nanofluid, heat transfer, carbon nanotube, heat sink

## Abstract

This study is to investigate the magnetohydrodynamic (MHD) stagnation point flow and heat transfer characteristic nanofluid of carbon nanotube (CNTs) over the shrinking surface with heat sink effects. Similarity equations deduced from momentum and energy equation of partial differential equations are solved numerically. This study looks at the different parameters of the flow and heat transfer using first phase model which is Tiwari-Das. The parameter discussed were volume fraction nanoparticle, magnetic parameter, heat sink/source parameters, and a different type of nanofluid and based fluids. Present results revealed that the rate of nanofluid (SWCNT/kerosene) in terms of flow and heat transfer is better than (MWCNT/kerosene) and (CNT/water) and regular fluid (water). Graphically, the variation results of dual solution exist for shrinking parameter in range $\lambda_{c}<\lambda \leq -1$ for different values of volume fraction nanoparticle, magnetic, heat sink parameters, and a different type of nanofluid. However, a unique solution exists at −1<λ<1, and no solutions exist at $\lambda <\lambda_{c}$ which is a critical value. In addition, the local Nusselt number decreases with increasing volume fraction nanoparticle when there exists a heat sink effect. The values of the skin friction coefficient and local Nusselt number increase for both solutions with the increase in magnetic parameter. In this study, the investigation on the flow and heat transfer of MHD stagnation point nanofluid through a shrinking surface with heat sink effect shows how important the application to industrial applications.

## 1. Introduction

Currently, nanofluid plays an important role in heat transfer enhancement. This is due to the efficiency of heat transfer, and it is useful in most components such as heat exchanger, electronic devices, and any equipment that involve on heat transfer rate. Conventional heat transfer fluid or base fluid such as water, kerosene, oil, and ethylene glycol have a low heat transfer rate due to poor thermal conductivity. Therefore, this shortcoming of heat transfer performance can be overcome by adding a single type of nanosized particle into base fluid. That is why nanofluid research has still been relevant in engineering and industrial application until today. Initially, study about heat transfer characteristics of nanofluid is reviewed [1]. It already mentions that convective heat transfer can be enhanced passively by enhancing thermal conductivity of the fluid. Next, Asirvatham et al. [2] investigated convective heat transfer of nanofluid with correlations. N. Kumar et al. [3] studied on nanofluid application for heat transfer in a microchannel. Numerical study of convective heat transfer of nanofluid is reviewed by Vanaki et al. [4]. It indicated that effective thermal conductivity and viscosity of nanofluid are predicted by considering the effect of volume fraction, particle shape, particle size, nanofluid temperature, and Brownian diffusion. Han et al. [5] conducted the experimental study of heat transfer enhancement using nanofluid in a double-tube heat exchanger. They concluded that heat transfer at boundary layer increases significantly with the addition of nanoparticles as constant bombarding of nanosized particle transfers much of the heat from the boundary to the mainstream fluid, thus increasing the heat transfer effect and Nusselt number. Furthermore, Chiam et al. [6] presented the numerical study of nanofluid heat transfer for different tube geometries. They mentioned that the convective heat transfer coefficient is strongly dependent on the surface of the solid, thermophysical properties of coolant, and the type of flow. Ahmadi and Willing [7] studied the heat transfer measurement in water based nanofluid. The study about flow and heat transfer behavior of nanofluid in microchannels is investigated by Bowers et al. [8]. They mentioned that the nanoparticles need to be as stable as possible to avoid clogging and sedimentation within heat transfer equipment. The study of Buschmann et al. [9] about the correct interpretation of nanofluid convective heat transfer has proven that the heat transfer enhancement provided by nanofluid equals the increase in the thermal conductivity of the nanofluid as compared to the base fluid that is independent of the nanoparticle concentration or material.

This study also involved the stagnation flow toward the shrinking sheet is already conducted by [10], which described the fluid motion near the stagnation region, which exists on all solid bodies moving in a fluid. This region encounters the highest pressure, heat transfer, and rates of mass deposition. Bhatti et al. [11] conducted the numerical simulation of fluid flow over a shrinking porous sheet by successive linearization method. This study confirmed the existence of a dual solution for shrinking sheet, while for the stretching case, the solution is unique. Soid et al. [12] investigated the axisymmetric stagnation-point of second-order velocity slip. It mentions that the value of skin friction coefficient being zero when λ = 1, because the fluid and the solid surface which move in the same velocity, and thus, there is no friction at the fluid-solid interface. However, there is a heat transfer at the surface, even though no friction occurred. This happens because of the temperature difference between the fluid and the solid surface. Dash et al. [13] presented the numerical approach to boundary layer stagnation-point flow past a stretching/shrinking sheet. It explained that the striking feature of the observation is that the shrinking of the boundary surface overrides the resistive effect of the electromagnetic force and sustains a backflow. Tasawar Hayat et al. [14] considered the inclined magnetic field and heat source/sink aspects in flow of nanofluid with nonlinear thermal radiation and examined the numerical simulation for melting heat transfer and radiation effects in stagnation point flow of carbon-water nanofluid [14].

The entropy generation on MHD flow and convective heat transfer in a porous medium of exponentially stretching surface saturated by nanofluids [15]. The study indicated that there is no viscous effect at the exterior to the boundary layer, and therefore, the pressure distribution can be obtained through the Euler form of the momentum equation. The thermal boundary layer in stagnation-point flow past a permeable shrinking sheet with variable surface temperature was studied by Uddin and Bhattacharyya [16]. The stagnation point flow of a micropolar nanofluid past a circular cylinder with velocity and thermal slip was explored by Abbas et al. [17]. The hydromagnetic unsteady slip stagnation flow of nanofluid with suspension of mixed bioconvection was investigated by R. Kumar et al. [18]. They discussed that region λ = 0 depicts that these forces are of equal magnitude. Mustafa et al. [19] considered an analytical solution of least square method. It is observed that the range of the dual solutions become larger by enhancing the effects of magnetic parameter. Al-Amri and Muthtamilselvan [20] examined that the stagnation point flow of nanofluid containing micro-organisms. Anuar et al. [21] investigated the MHD flow past a nonlinear stretching/shrinking sheet in carbon nanotubes including stability analysis. It is clearly that when $\lambda <\lambda_{c}$, the solution does not exist because boundary layer separation occurs that causes the boundary layer equation to be invalid. The finding was also similar found by [22]. Furthermore, the study of stretching/shrinking sheet of magnetic nanofluid might be helpful for researchers to study the stability of working fluid [23,24].

In a nutshell, this article is considered MHD stagnation point nanofluid flow and heat transfer of carbon nanotube over a shrinking surface with heat sink effect. The molecular interaction of SWCNT considering the stagnation point and heat sink in different based fluids has few studies discussed by many researchers. Due to high thermal conductivity of CNT and the potential to improve heat transfer, thus, this study also considers water and kerosine as a base fluid and carbon nanotube (CNT) including single-wall carbon nanotube (SWCNT) and multiwall carbon nanotube (MWCNT) as a nanoparticle. The results were obtained in numerical, and they are presented in form of graphs and tables to describe the behavior of this study and are compared with previously published results to achieve a good agreement.

## 2. Methodology

**Figure 1:**
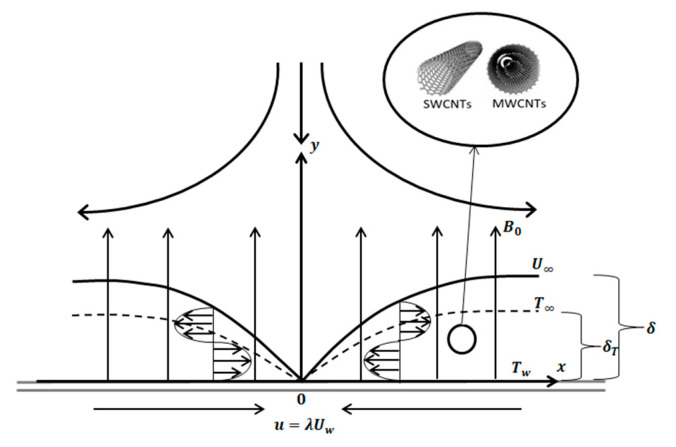
MHD stagnation flow of nanofluid past shrinking sheet.

Let continuity Equation (1), momentum Equation (2), and energy Equation (3) be

(1) $$\frac{\partial u}{\partial x}+\frac{\partial \nu }{\partial y}=0$$

(2) $$u\;\frac{\partial u}{\partial x}+\nu \frac{\partial u}{\partial y}=\frac{\mu_{nf}}{\rho_{nf}}\left[\frac{\partial^{2}u}{\partial y^{2}}\right]+U_{\infty }\frac{\partial U_{\infty }}{\partial x}+\frac{\sigma B_{0}^{2}}{\rho_{nf}}\left(U_{\infty }-U\right)$$

(3) $$u\;\frac{\partial T}{\partial x}+\nu \frac{\partial T}{\partial y}=\frac{k_{nf}}{{\left(\rho C_{\rho }\right)}_{nf}}\;\frac{\partial^{2}T}{\partial y^{2}}+\frac{Q}{{\left(\rho C_{\rho }\right)}_{nf}}\;\left(T-T_{\infty }\right)$$

given that the boundary condition of governing equation is given as follow:

(4) $$u=U_{w}=cx,\;\nu =0,\;T=T_{w}\;\mathrm{as}\;y=0\;u=U_{\infty }=ax,\;T=T_{\infty }\;\mathrm{as}\;y\to \infty$$

where (u, ν) is velocity component along *x* and *y* axis, respectively. $U_{w}$ as velocity wall, $U_{\infty }$ is free flow velocity, *T* is temperature, and $T_{\infty }$ is ambient temperature. (*a*, *c*) is a positive constant which refer to stretching/shrinking strength where stretching case is *c* > 0 whereas shrinking case is *c* < 0, and $T_{w}$ is temperature wall. All nomenclature in Equations (1)–(3) are illustrated in Nomenclature. Figure 1 show the working flow and heat transfer for MHD stagnation point with shrinking surface. For this case, the assumptions of impermeable wall, uniform nanoparticles size, agglomeration effect, and viscous dissipation are neglected. The base fluid and the nanoparticles are similarly considered to be in thermal equilibrium in the Tiwari–Das nanofluid model, with no-slip between them. Heat transport, convection, and the heat sink effect are all accounted for in energy equations. This study focuses on laminar flow for the working liquid; hence, it is expected that large velocity gradient existed, and therefore, the viscous dissipation term in Equation (3) is omitted.

The nanoparticle used is considered in this problem study to discover the behavior the MHD flow and heat transfer of nanofluid. Therefore, Table 1 shows that the effective thermophysical properties of nanofluid needed to explain the nanofluid model. The empirical shape factor is set *n* = 3/*m* = 3 where *m* is referred to ideal spherical shape. Table 2 shows the thermophysical properties used by [15,21] for different nanoparticle and fluid selected in this problem study. In this method, the authors investigate the outcome for flow and heat transfer simultaneously using bvp4c. Hence, the influence of carbon nanotube aspect ratio is neglected.

Furthermore, stagnation point flow in this problem study was also considered. This is because stagnation point flow produced on static surface either stretching or shrinking. Therefore, the similarity solution for the problem of MHD stagnation point flow in nanofluid and heat transfer over shrinking surface with heat sink effect is given as follows:

(5) $$\psi =\sqrt{\nu_{f}\;ax}\;f\left(\eta \right),\;\;\eta =\sqrt{\frac{a}{\nu_{f}}}\;y,\;\;\theta \left(\eta \right)=\frac{T-T_{\infty }}{T_{w}-T_{\infty }}$$

with ψ being a stream function that is defined as *u* and ν. Thus,

(6) $$u=\frac{\partial \psi }{\partial y}=axf^{\prime }\left(\eta \right),\;\;\nu =-\frac{\partial \psi }{\partial x}=-\sqrt{a\nu_{f}\;}f\;\left(\eta \right)$$

where (′) shows the differentiation with respect to η. Thus, the mathematical model in form of ordinary differential equation (ODE) is stated as follows:

(7) $$\frac{1}{{\left(1-\varphi \right)}^{2.5}}f^{\prime \prime \prime }\left(\eta \right)-\frac{\rho_{nf}}{\rho_{f}}\left(f^{\prime 2}\left(\eta \right)-f\left(\eta \right)f^{\prime \prime }\left(\eta \right)-1\right)+M\left(1-f^{\prime }\left(\eta \right)\right)=0\;\frac{1}{Pr}\;\frac{k_{nf}}{k_{f}}\theta \prime \prime \left(\eta \right)+\left(\left(1-\varphi \right)+\varphi \frac{{\left(\rho C_{p}\right)}_{s}}{{\left(\rho C_{p}\right)}_{f}}\right)f\left(\eta \right)\theta^{\prime }\left(\eta \right)+\varepsilon \theta \left(\eta \right)=0$$

subject to boundary condition:

(8) $$f^{\prime }\left(\eta \right)=\lambda ,\;f\left(\eta \right)=0,\;\theta \left(\eta \right)=1\;\mathrm{as}\;\eta =0\;f^{\prime }\left(\eta \right)=1,\;\theta \left(\eta \right)=0\;\mathrm{as}\;\eta \to \infty$$

where (′) represent differentiation with respect to η, φ as volume fraction nanoparticle, $\rho_{nf}$ is density of nanofluid, $\rho_{f}$ is density of fluid, M = $\sigma B_{0}^{2}\;/$$a\rho_{f}$ is magnetic parameter, Pr = $\nu_{f}$/$\alpha_{f}$ as Prandtl number, $\left(\rho C_{p}\right){\;}_{nf}$ is specific heat capacity of nanofluid, $\left(\rho C_{p}\right){\;}_{f}$ is specific heat capacity of fluid, and ε = *Q*/$a\left(\rho C_{p}\right){\;}_{f}$ is heat sink/source parameter. As for the boundary condition, λ = *c/a* is referred to stretching/shrinking strength or velocity parameter, where λ>0 is stretching case, whereas λ<0 is shrinking case. The interpretation of the physical quantity considered in the study are local skin friction coefficient, $C_{f}$ and local number Nusselt, $Nu_{x}$ which can be given as follows:

(9) $$C_{f}=\frac{\tau_{w}}{\rho_{f}U_{\infty }^{2}},\;\;\;\;Nu_{x}=\frac{xq_{w}}{k_{f}\left(T_{w}-T_{\infty }\right)}$$

with shear stress, $\tau_{w}$ and heat flux, $q_{w}$ which can be defined as follows:

(10) $$\tau_{w}=\mu_{nf}{\left(\frac{\partial u}{\partial y}\right)}_{y=0},\;\;\;q_{w}=-k_{nf}{\left(\frac{\partial T}{\partial y}\right)}_{y=0}$$

By using the Reynold number coefficient, $Re_{x}$ = $U_{\infty }x$/$\upsilon_{f}$, thus local skin friction coefficient, $Re_{x}{}^{1/2}C_{f}$ and local number Nusselt, $Re_{x}{}^{-1/2}Nu_{x}$ can be stated as follows:

(11) $$\;Re_{x}{}^{1/2}C_{f}=\frac{1}{{\left(1-\varphi \right)}^{2.5}}f\prime \prime \left(0\right)\;Re_{x}{}^{-1/2}Nu_{x}=-\frac{k_{nf}}{k_{f}}\theta \prime \left(0\right)$$

In this study, we investigate the MHD laminar flow where the condition of the flow is assumed stable. Hence, we do not consider the stability analysis for the first and second solutions. Throughout Equations (1)–(11), the Tiwari–Das nanofluid model did not consider the mass transfer of carbon nanotubes. However, the formulation of nanoparticle volume fraction is the advantage of this model to explain the interaction of nanoparticle with working fluids. For this case, the flow and heat transfer of nanofluid are produced and present numerically. Although recent studies [25,26] investigated the nonuniform dispersion of nanoparticle, the Tiwari–Das would be able to measure the flow and heat transfer of nanofluids. However, the limitation of this method is not being able to measure the Brownian motion and thermophoresis interaction between nanoparticles. As described by [27], Brownian dynamic might be able to measure the nonuniform dispersion of nanoparticle.

## 3. Results

The numerical solutions from the governing ordinary differential equation for flow and energy with its boundary condition were solved by using bvp4c solver in MATLAB software. This solver is based on three-stage collocation at Lobatto point which means the three-stage Lobatto IIIA method. Lobatto IIIA methods can be very efficient for the numerical solution of nonlinear stiff systems (12). These numerical solutions are analyzed and presented in tables and graphs for discussing the behavior of flow and heat transfer of this boundary layer model when including a few parameters. This study is conducted by adding nanoparticle volume fraction of CNT from 0 to 0.2 in range 0≤φ≤0.2 into base fluid which are water and kerosene selected. Besides that, the parameters values which varied on λ are φ, M, ε, and the nanofluid selected as well as this parameter value varied in region $\lambda_{c}<\lambda <1$. This is because second solution is discovered in range λ<−1 that shows shrinking case and meet the requirements of the study conducted. Next, few numerical results produced of $f^{\prime \prime }\left(0\right)$ are compared with previous results that are shown in Table 3.

Based on Table 3, it was found that each numerical result is compared to achieve a good agreement when the nanoparticle is not considered in base fluid. The numerical result obtained is compared with previous result to ensure mathematical model developed and solver method used are valid before numerical solution when the set up for the parameter selected is produced. However, the present comparison results numerically for the $f^{\prime \prime }\left(0\right)$ value with [28] the results being slightly different when volume fraction nanoparticle, and φ is added in base fluids, which are 0.1 and 0.2. In this study, the range of nanoparticle volume fraction with a range of 0–0.2 is chosen based on study by [29], whereas the range of magnetic parameters is set between 0 and 0.2. The numerical result is different because the nanoparticle used is different in base fluid. The problem in this study with the nanoparticle selected is the carbon nanotube (CNT), whereas for Bachok’s (2011) study, it was copper. Therefore, different types of nanoparticles in same base fluid have different thermophysical properties and of course give the different numerical result of $f^{\prime \prime }\left(0\right)$ and $-\theta^{\prime }\left(0\right)$.

**Figure 2:**
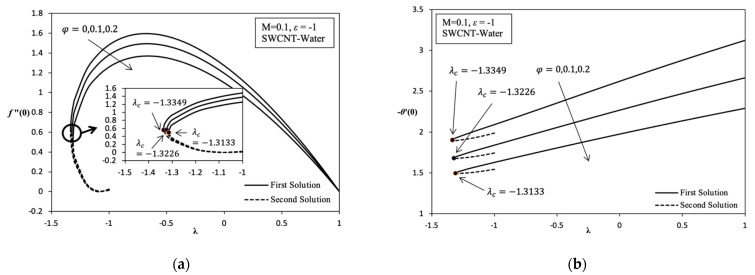
Variation on λ with various values of φ in SWCNT-water when *M* = 0.1 and ε=−1: (**a**) Variation on *f*″ (0); (**b**) Variation on −θ ′ (0).

**Figure 3:**
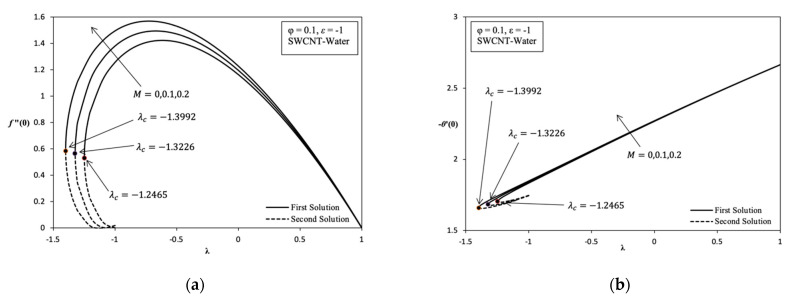
Variation on λ with various values of M in SWCNT-water when φ = 0.1 and ε=−1: (**a**) Variation on *f*″ (0); (**b**) Variation on −θ ′ (0).

**Figure 4:**
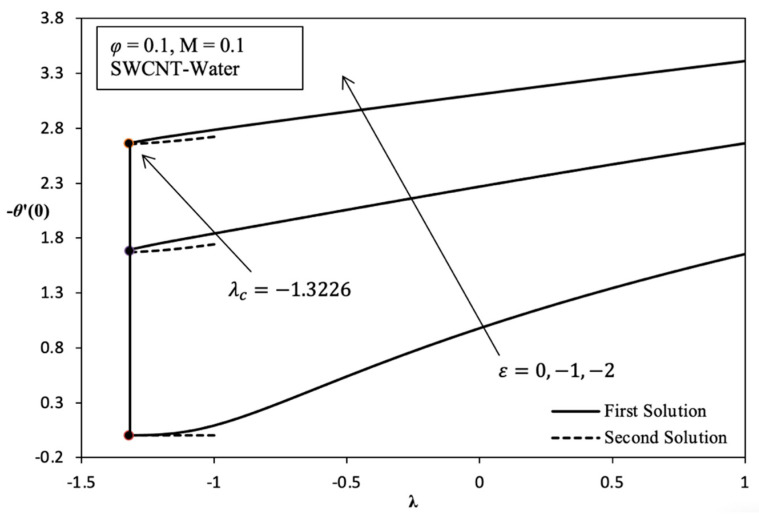
Variation of −θ ′(0) versus λ with various values of ε in SWCNT-water when φ = 0.1 and *M* = 0.1.

**Figure 5:**
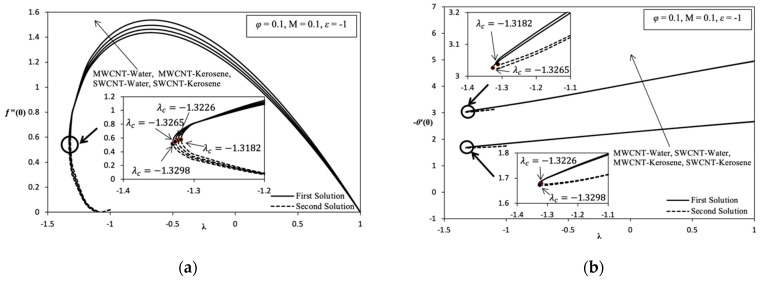
Variation on λ with various values of nanomaterial when φ = 0.1, M = 0.1, and ε=−1: (**a**) Variation on *f*″ (0); (**b**) Variation on −θ ′ (0).

**Figure 6:**
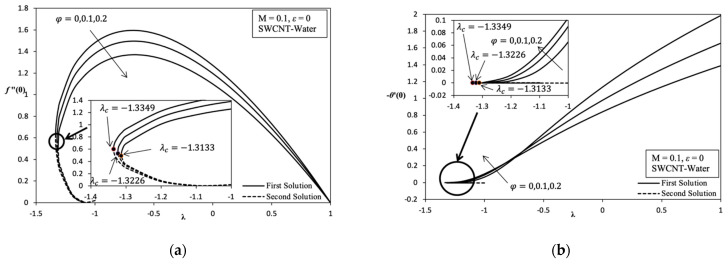
Variation on λ with various values of φ  in SWCNT-water when M= 0.1: (**a**) Variation on *f*″ (0); (**b**) Variation on −θ ′ (0).

Figure 2, Figure 3, Figure 4, Figure 5 and Figure 6 shows the existence of a dual solution clearly. This solution can be observed in region $\lambda_{c}\leq \lambda \leq 1$ and the existence of unique solution at point $\lambda_{c}$ = λ where $\lambda_{c}$ is critical point as well as at region λ>−1. Based on region produced $\lambda_{c}\leq \lambda \leq 1$, the mathematical model developed has the potential to describe the behavior of MHD nanofluid over the different parameter values. A numerical solution does not exist when in the region $\lambda_{c}>\lambda$. Thus, this case shows the incompatibility mathematical model in the region or not being able to easily understand the boundary layer separation and boundary layer approximation are physically cannot be realized. The discussion of this problem study is continued with addition of carbon nanotube (CNT), which is a single-wall carbon nanotube (SWCNT) on local skin friction coefficient, $Re_{x}{}^{1/2}C_{f}$ and local Nusselt number, $Re_{x}{}^{-1/2}Nu_{x}$. Figure 2a indicates the change in trend $Re_{x}{}^{1/2}C_{f}$ which can be referred to as $f^{\prime \prime }\left(0\right)$ on the variation value of volume fraction nanoparticle of SWCNT, φ when stretching/shrinking surface. Stretching/shrinking case that shows in first solution describe the reduction of $Re_{x}{}^{1/2}C_{f}$ when the value of φ increases from 0 to 0.2. Although nanofluid becomes more viscous, it is still not enough to achieve the enhancement of $Re_{x}{}^{1/2}C_{f}$ when the value of φ increases. Therefore, the enlargement in momentum boundary layer thickness, δ which coincides with the increase in value of φ that causes the decrease in $Re_{x}{}^{1/2}C_{f}$ when the value of φ increases.

Furthermore, Figure 2a, Figure 3a, Figure 5a and Figure 6a described that when λ=1 indicates a value of $Re_{x}{}^{1/2}C_{f}$ is zero. This means the velocity fluid flow of stagnation point is equivalent with a velocity wall at rate λ=1, which is due to there being no friction that occurs on surface. The point at (1, 0) is also known as the transition point. The second solution shows that the value of $Re_{x}{}^{1/2}C_{f}$ slightly increases when the value of φ increases in momentum with the boundary layer thickness slightly thinning and slightly increasing in skin friction along the shrinking case. The dual solution in Figure 2b shows the reduction in local Nusselt number, $Re_{x}{}^{-1/2}Nu_{x}$ which can be referred to as $-\theta^{\prime }\left(0\right)$, when the value of φ increases from 0 to 0.2. There is a significant reduction in the value of $Re_{x}{}^{-1/2}Nu_{x}$ along the shrinking case because of the existence of the heat sink effect, ε<0 given that the problems study also considers the parameters, ε to discover various type of behaviors of this model. Consequently, thermal boundary layer thickness $\delta_{T}$ becomes thick when the value of φ increases and the temperature gradient decreases. Thus, this statement proves that when the numerical result with the change value of φ from 0 to 0.2, which is produced in the variation of λ with heat sink effect, is neglected, ε=0. When this is performed, there is an increase in the value of $Re_{x}{}^{-1/2}Nu_{x}$ along the shrinking case that shows in Figure 6b, and it increases temperature gradient, and the thermal boundary layer thickness becomes thin. However, the value of $Re_{x}{}^{1/2}C_{f}$ continues to decreases, thus increasing the value of φ that is shown in Figure 6a. This proves that nanofluids have a better heat enhancement compared with fluid φ=0. Because of the existence of heat sink effect, ε<0 in this model further inhibits the heat transfer rate of increasing φ. Thus, it is worth noting that if the parameter φ is applied, only a few values of φ used in the base fluid are enough for heat transfer enhancement.

In addition, Figure 3a also highlights the increasing value of $Re_{x}{}^{1/2}C_{f}$ along with the increasing value of magnetic parameter, M, in the stretching/shrinking case. This is happens because of the Lorentz force J×B, which is equivalent to drag or the viscosity force acting on the surface. However, it is opposed by fluid flow. It can significantly increase the shear stress on the shrinking surface. Thus, the momentum boundary layer thickness decreases with the increasing value of M. The second solution described the decreasing value of $Re_{x}{}^{1/2}C_{f}$ when M increases from 0 to 0.2. The dual solution in Figure 3b shows a slight increase in $Re_{x}{}^{-1/2}Nu_{x}$ when the value of M is higher from 0 to 0.2. Then, the heat transfer rate increases because of the thermal boundary layer thickness becoming thin, and it causes a temperature gradient increase. Thus, the existence of MHD stagnation flow of nanofluid gives a good impact in terms of heat transfer enhancement in application terms, for example, the heat exchanger process and cooling system.

**Figure 7:**
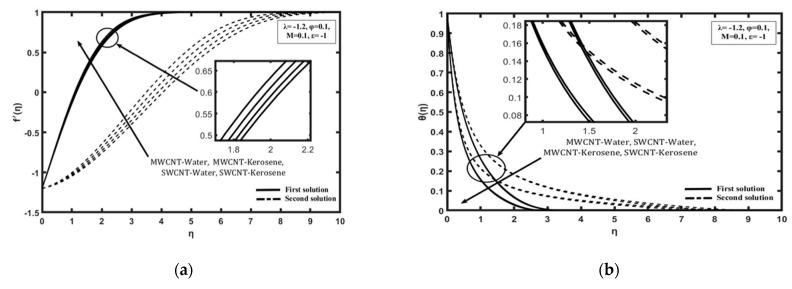
Various CNT and based fluid when M= 0.1, λ = − 1.2, φ = 0.1, and ε=−1: (**a**) Velocity profile; (**b**) Temperature profile.

Moreover, it was found that the increasing value of $Re_{x}{}^{-1/2}Nu_{x}$ goes along with increasing value of heat sink parameter ε from 0 to −2 as shown in Figure 4. As far as we know, the value of $Re_{x}{}^{1/2}C_{f}$ does not show any change of parameter ε or, in other words, is uniform because this parameter does not depend on momentum. This indicates that the thermal boundary layer thickness decreases when ε<0 increases, causing the temperature gradient to be higher. Therefore, heat transfer enhancement is improving with the increasing heat sink effect, which is commonly found in application cooling systems on electronic devices. Figure 5a,b shows the variation of $Re_{x}{}^{1/2}C_{f}$ and $Re_{x}{}^{-1/2}Nu_{x}$, respectively, on the different of nanofluids selected for solving the problem study which are SWCNT-kerosene, SWCNT-water, MWCNT-kerosene, and MWCNT-water. Based on Figure 5a, it is indicated that the value of $Re_{x}{}^{1/2}C_{f}$ of SWCNT-kerosene is the highest compared with the different nanofluids followed by SWCNT-water, MWCNT-kerosene, and MWCNT-water. This is because the SWCNT nanoparticle is better than MWCNT, as well as kerosene being higher than the value of the Prandtl number than water which is, respectively, around 21 and 6.2. Thus, the momentum boundary layer thickness on the different of nanofluid is followed by a thick layer, which is made up of MWCNT-water, MWCNT-kerosene, SWCNT-water, and SWCNT-kerosene. Based on Figure 5b, it was proven that SWCNT-kerosene nanofluid have shown the highest value of $Re_{x}{}^{-1/2}Nu_{x}$ followed by MWCNT-kerosene, SWCNT-water, and MWCNT-water. The MWCNT-water nanofluid is among the most deteriorating nanofluid in terms the value of $Re_{x}{}^{1/2}C_{f}$ and $Re_{x}{}^{-1/2}Nu_{x}$, which cause the momentum and thermal boundary layer thickness to become thick, thus affecting the heat transfer process. Hence, this conclusion is justified based on the velocity and temperature profile produced in Figure 7a,b.

Overall, it is indicated that the variation of $Re_{x}{}^{1/2}C_{f}$ and $Re_{x}{}^{-1/2}Nu_{x}$ in each parameter, which are φ, M, ε, and the different nanofluids are able to provide their own critical value. Based on Figure 2, Figure 3, Figure 4, Figure 5 and Figure 6 for the variation result, it is shown that the different value of λ in the more shrinking case causes a significant increase in the value of $Re_{x}{}^{1/2}C_{f}$ while significantly decreasing the value of $Re_{x}{}^{-1/2}Nu_{x}$.

## 4. Conclusions

This research is about MHD stagnation point flow and heat transfer of nanofluids over a shrinking surface with the heat sink effect being analyzed numerically and discussed in detail in this paper. It was found that the involved parameters such as magnetic parameter, heat sink effect, different types of nanoparticles and base fluids significantly affect the flow and heat transfer. It was found that a decrease in both velocity and temperature was observed with an increase in the volume fraction nanoparticle of the carbon nanotube parameter when the heat sink effect existed, ε<0. When there is no heat sink effect, ε=0 for the shrinking case, and the velocity decreases while the temperature increases. Dual solutions exist up to a certain range of the shrinking parameter. This study noticed that both the skin friction coefficient and local Nusselt number increased with an increase in the Magnetic parameter. It was also observed that the values of f″(0) were unchanged while the values of −θ′(0) increased with the increase in heat sink parameter, ε<0. Based on the type of nanofluid, SWCNT-kerosene has the highest skin friction coefficient and local Nusselt number. Through the literature conducted for a similar problem, it was concluded that SWCNT-kerosene nanofluid tends toward heat transfer enhancement more than other nanofluid types. Moreover, nanofluid is better than conventional fluid because of local Nusselt number, with −θ′(0) both increasing more than conventional fluid when the heat sink effect does not exist, ε=0 and the velocity parameter is near to critical value of $\lambda_{c}$ or shrinking case, λ<0, although the skin friction coefficient, f″(0) decreases.

## Figures and Tables

**Table 1:** The effective thermophysical properties of nanofluid.

Thermophysical Properties	Nanofluid CNT-Water (*s* = CNT: *n* = 3)
Density ($kg/m^{3}$)	$\rho_{nf}=\left(1-\varphi \right)\rho_{f}+\varphi \rho_{s}$
Heat capacity (J/K)	${\left(\rho C_{\rho }\right)}_{nf}=\left(1-\varphi \right){\left(\rho C_{\rho }\right)}_{f}+\varphi {\left(\rho C_{\rho }\right)}_{s}$
Viscosity ($Ns/m^{-2}$)	$\mu_{nf}=\frac{\mu_{f}}{{\left(1-\varphi \right)}^{2.5}}$
Thermal conductivity (W/Km)	$\frac{k_{nf}}{k_{f}}=\frac{k_{s}+\left(n-1\right)k_{f}-\left(n-1\right)\varphi \left(k_{f}-k_{s}\right)}{k_{s}+\left(n-1\right)k_{f}+\varphi \left(k_{f}-k_{s}\right)}$

**Table 2:** Thermophysical properties of nanoparticle, nanoparticle, and base fluid [15,21].

Thermophysical Properties	Base Fluid		Nanoparticle	
	Water Pr = 6.2	Kerosine Pr = 21	SWCNT	MWCNT
ρ ($kg/m^{3}$)	997.1	783	2600	1600
$C_{p}\left(J/kg\;K\right)$	4179	2090	425	796
k(W/mK)	0.613	0.145	6600	3000

**Table 3:** Comparison several of numerical result f″(0) when stretching/shrinking case and *M* =ε=0 with the change of φ and λ in nanofluid.

φ	λ	$f^{\prime \prime }\left(0\right)$					
		Present Result		Bachok et al. [28]		Wang [29]	
		CNT-Water		Cu-Water		Water	
		First Solution	Second Solution	First Solution	Second Solution	First Solution	Second Solution
0	2	−1.887306668		−1.887307		−1.88731	
	1	0		0		0	
	0.5	0.71329495		0.713295		0.7133	
	0	1.232587647		1.232588		1.232588	
	−0.5	1.495669739		1.49567		1.49567	
	−1	1.328816861	0	1.328817	0	1.32882	0
	−1.2	0.932473188	0.233649469	0.932473	0.23365		
0.1	2	−1.78244491		−2.217106			
	1	0		0			
	0.5	0.673663145		0.83794			
	0	1.164103115		1.447977			
	−0.5	1.412567954		1.757032			
	−1	1.254985673	0	1.561022	0		
	−1.2	0.880663529	0.220667553	1.095419	0.274479		
0.2	2	−1.641498824		−2.298822			
	1	0		0			
	0.5	0.620393516		0.868824			
	0	1.072052147		1.501346			
	−0.5	1.300869722		1.821791			
	−1	1.155748188	0	1.618557	0		
	−1.2	0.811025466	0.20321847	1.135794	0.284596		

## Data Availability

Not applicable.

## Nomenclature

Symbols		Greek Symbols	
*c*, *a*	Positive constant	ε	Heat sink/source parameter
$B_{0}$	Magnetic field (induced)	σ	Electrical conductivity
$C_{f}$	The coefficient of skin friction	$\mu_{nf}$	Nanofluid viscosity
M	Magnetic	${\left(\rho C_{p}\right)}_{nf}$	Nanofluid heat capacity
$Nu_{x}$	Nusselt number;	ρ	Density
Pr	Prandtl number	λ	Stretching/shrinking parameter
Rd	Thermal radiation	*k*	Thermal conductivity
$Re_{x}$	Reynold number	φ	Volume fraction of CNT
*T*	Fluid temperature	Subscript	
*u,v*	Velocity component (*x*- and *y*-axes)	*nf*	Nanofluid
$U_{w}$	Velocity (stretching/shrinking sheet)	*f*	Base fluid
		*s*	Solid
	Superscript	*c*	Critical
(′)	Prime denotes differentiation with respect to *η*	∞	Far field condition/ambient
		*w*	Surface condition
